# Effects of Rutin on Wound Healing in Hyperglycemic Rats

**DOI:** 10.3390/antiox9111122

**Published:** 2020-11-13

**Authors:** Li-You Chen, Chien-Ning Huang, Chih-Kai Liao, Hung-Ming Chang, Yu-Hsiang Kuan, To-Jung Tseng, Kai-Jung Yen, Kai-Lin Yang, Hsing-Chun Lin

**Affiliations:** 1 Department of Anatomy, School of Medicine, College of Medicine, Chung Shan Medical University, Taichung City 40201, Taiwan; payueric@gmail.com (L.-Y.C.); ckliiao37@csmu.edu.tw (C.-K.L.); tjtsenng@csmu.edu.tw (T.-J.T.); kensky97856@gmail.com (K.-J.Y.); kevin31386587@gmail.com (K.-L.Y.); 2 Department of Medical Education, Chung Shan Medical University Hospital, Taichung City 40201, Taiwan; 3 Department of Internal Medicine, Division of Endocrinology and Metabolism, Chung Shan Medical University Hospital, Taichung City 40201, Taiwan; cshy049@gmail.com; 4 Institute of Medicine, College of Medicine, Chung Shan Medical University, Taichung City 40201, Taiwan; 5 Department of Anatomy and Cell Biology, School of Medicine, College of Medicine, Taipei Medical University, Taipei City 40201, Taiwan; taiwanzoo@gmail.com; 6 Department of Pharmacology, School of Medicine, Chung Shan Medical University, Taichung City 40201, Taiwan; kuanyh@csmu.edu.tw; 7 Department of Pharmacy, Chung Shan Medical University Hospital, Taichung City 40201, Taiwan; 8 Department of Nutrition, Chung Shan Medical University, Taichung City 40201, Taiwan; 9 Department of Nutrition, Chung Shan Medical University Hospital, Taichung City 40201, Taiwan

**Keywords:** rutin, hyperglycemia, wound healing, antioxidant, anti-inflammatory

## Abstract

Long-term poor glycemic control negatively affects macrovascular and microvascular diseases, as well as wound restoration. Buckwheat is a good source of rutin (quercetin-3-O-rutoside) and has benefits in regulating blood sugar. This study was to evaluate the antioxidant and anti-inflammatory effects of rutin on wound healing in streptozotocin-induced hyperglycemic rats. Eighteen male Wistar rats were randomly divided into three groups: normal (NDM), hyperglycemic (DM), and hyperglycemic with rutin (DMR). After induction of hyperglycemia for 2 days, a 15 × 15 mm wound was induced on the back of each rat. Intraperitoneal injection of rutin significantly ameliorated diabetes-induced body weight loss and improved metabolic dysfunctions of hyperglycemic rats. Based on appearance and histopathological staining, rutin promotes wound healing and inhibits production of inflammatory cells. The immunoblotting data indicated that rutin promotes production of antioxidant enzymes induced by nuclear factor erythroid 2-related factor 2 (NRF2), inhibits the expression of matrix metalloproteinases (MMPs) regulated by NF-κB, and decreases the expression of vascular endothelial growth factor (VEGF). It also promotes the expression of neurogenic-related protein (UCH-L1). The aforementioned results indicated that rutin reduces oxidative stress and inflammatory response in hyperglycemic rats, promoting wound healing and subsequently reducing the risk of wound ulcers.

## 1. Introduction

According to an International Diabetes Federation (IDF) report published in 2019, diabetes has become one of the most important public health issues globally [1]. Diabetes is a complex chronic disease that causes glucose induction, insulin secretion disorders, autoimmune-mediated beta cell destruction, or inadequate compensation of insulin secretion for insulin resistance. Complications such as macrovascular and microvascular diseases, neuropathy, and slow wound healing are common in diabetic patients [2]. Both diabetes and wounds can induce oxidative stress, especially hyperglycemia, which enhances the generation of free radicals and reduces anti-oxidation capabilities. Free radicals destroy the ability of β-cells to secrete insulin and increase the incidence of diabetes complications [3,4]. In addition, long-term exposure of cells and tissues to the hyperglycemic environment affects the metabolism of proteins and lipids, induces the production of reactive oxygen species (ROS), promotes inflammation, increases the risk of wound ulcers, and delays wound healing [4]. Therefore, oxidative stress and diabetes are mutually causal, resulting in a vicious cycle.

Diabetic foot ulcers (DFUs) often take a long time to heal and their recurrence rate is very high. About 25% of diabetic patients develop DFUs [5]. Hyperglycemia affects wound healing in diabetic patients, in addition to vascular disease and neuropathy. Severe DFUs may require amputation. The wound healing process is complex and consists of four consecutive and overlapping steps: hemostasis, inflammation, proliferation, and remodeling [6]. The repair process requires coordination of different cells, growth factors, and inflammatory cytokines [7]. During the healing phase, angiogenesis and matrix reorganization are regulated by growth factors, including vascular endothelial growth factor (VEGF), platelet-derived growth factor (PDGF), transforming growth factor (TGF), interleukin (IL), and tumor necrosis factor (TNF) [8,9]. Macrophages, neutrophils, and fibroblasts regulate the remodeling of extracellular junctional tissue and wound matrix by producing these factors [10]. Among them, the mobility of keratinocytes and fibroblasts and related enzymes of extracellular matrix reorganization play a key role in wound healing, especially matrix metalloproteinases (MMPs) and tissue inhibitors of matrix metalloproteinases (TIMPs) [6].

There is a close relationship between nutrition and oxidative stress. Phytochemicals, as anti-inflammatory drugs, have been studied for their potential in improving diabetic wound healing for many years [11]. Flavonoids found in natural plants are natural pigments of fruits and vegetables, which have beneficial effects on diabetes by improving glycemic control, lipid distribution, and antioxidant status. Buckwheat is a good source of rutin (quercetin-3-O-rutoside) and is found in buckwheat seeds, stems, leaves and flowers [12]. The content of rutin in different varieties and parts of buckwheat is also different. Taking buckwheat seeds as an example, the content of rutin is 0.05–1.35% (0.05–1.35 g per 100 g of dry seeds) [13]. Numerous studies have shown that rutin has anti-oxidant, anti-inflammatory, neuroprotective, nephroprotective, and hepatoprotective effects [14,15,16,17]. The mutual effects of oxidative damage, inflammation, and hyperglycemia are the main causes of delayed wound healing. Therefore, the purpose of this study was to evaluate the effects of rutin on wound healing in streptozotocin-induced hyperglycemic rats, especially its anti-oxidant and anti-inflammatory effects.

## 2. Materials and Methods

### 2.1. Chemicals

Rutin was purchased from Sigma-Aldrich (St. Louis, MO, USA). Based on previous studies, 100 mg/kg body weight of rutin was used in this animal experiment [18,19,20]. Streptozotocin (STZ) was also obtained from Sigma-Aldrich. All chemical reagents used in this study were of analytical grade.

### 2.2. Animal Feeding and Induction of Hyperglycemia

Eight-week-old male Wistar rats weighing 250–300 g each were obtained from BioLASCO (Taipei, Taiwan). They were housed in the Experimental Animal Center of Chung Shan Medical University, Taiwan, under a 12 h light–dark cycle at 25 ± 5 °C in a ventilated room. They were given ad libitum access to standard laboratory food and water. Intraperitoneal injection of STZ 80 mg/kg body weight was used to induce hyperglycemia [21]. This study was approved by the Institutional Animal Care and Use Committee (IACUC)—registration number and registration validity period: IACUC number: 2261/Valid from 1 January 2020 to 31 December 2020.

### 2.3. Animal Wound Healing

Rats were fasted overnight and then intraperitoneally injected with STZ (80 mg/kg). Two days after induction, hyperglycemia (fasting blood glucose level over 250 mg/dL) was confirmed and wound surgery was performed. The DMR group received an intraperitoneal injection of rutin (100 mg/kg) the next day after the operation. A 15 × 15 mm wound was induced on the back of each rat. The wound was rinsed daily with sterile saline. The rats were randomly divided into three groups (*n* = 6): (1) non-diabetes group (NDM): normal Wistar rats; (2) DM group (DM): induced hyperglycemia, without rutin; (3) DM + rutin group (DMR): induced hyperglycemia, with rutin (100 mg/kg body weight ip). Observation and evaluation of wound healing were carried out using Image J software (National Institutes of Health, Bethesda, MD, USA) [22]. Subsequently, the wound contraction rate was calculated according to the following formula [22]:

(Day N wound area − Day 0 wound area)/Day 0 wound area × 100%

### 2.4. Blood Samples

Blood samples were collected from the left ventricle with perfusion procedure and centrifuged at 1500× *g* for 20 min in a centrifuge. The concentrations of glucose, insulin, aspartate aminotransferase (AST), alanine aminotransferase (ALT), *alkaline phosphatase* (*ALP*), triglyceride (TG), cholesterol (CHO), and high-density (HDL) and low-density (LDL) lipoproteins in the serum were detected using commercially available reagents and instruments in accordance with the standard operating procedures recommended by the manufacturer.

### 2.5. Perfusion and Tissue Preparation

Before sacrifice, the rats were divided into two groups. One group received transcardiac perfusion for hematoxylin and eosin staining (H&E staining; Vector Laboratories), Masson trichrome staining, and immunohistochemical staining (IHC staining). Next, animals from each experimental group were deeply anesthetized by intraperitoneal injection of ketamine (100 mg/kg) and xylazine (10–13 mg/kg). Intracardiac perfusion was performed and tissue samples were fixed with 4% paraformaldehyde in 0.1% phosphate buffer (PB) (pH 7.4). After perfusion, the tissue samples were immersed in graded concentration of sucrose buffer (10–30%) and stored at 4 °C overnight. Continuous 30 mm thick slices of the wound were cut horizontally with a cryostat (CM3050S, Leica Microsystems, Wetzlar, Germany) the next day.

### 2.6. Histopathology and Staining

A stored biopsy sample from each group was washed with distilled water, then dehydrated with methanol. The samples were removed in xylene, embedded in paraffin in a hot air oven at 56 °C for 24 h, and cut into 5 μm thick tissue sections with a microtome. After dewaxing, staining with hematoxylin and eosin was carried out to observe the number of inflamed cells, angiogenesis, epithelial formation, and arrangement of extracellular matrix. Masson trichrome staining was used to explore the growth and arrangement of collagen fibers in experimental rats.

### 2.7. Immunohistochemical Staining

To eliminate endogenous peroxidase activity, the sections were immersed in 0.01 M PBS containing 3% H_2_O_2_/methanol solution for 1 h and washed with 0.01 M PBS (pH 7.4). Following 3 rinses in PBS, sections were incubated in the blocking medium containing 0.1% Triton X-100, 3% normal goat serum and 2% bovine serum albumin for 1 h to block nonspecific binding. Then, they were reacted with primary antibody NFκB (p65) (1:200, Santa Cruz, sc-8008), TNF-α (1:200, Santa Cruz, sc-52746), IL-1β (1:200, Santa Cruz, sc-52012), IL-6 (1:500, Bioss bs-0782R-TR), MMP-9 (1:200, Santa Cruz, sc-13520) and VEGF (1:200, Santa Cruz, sc-7269) at 4 °C for 48 h. After washing three times with 0.01 M PBS (pH7.4), the sections were placed in 1:200 secondary antibody at room temperature for 2 h. After washing with 0.01M PBS, the sections were immersed in 1:100 concentration of avidin–biotin complex for 1 h at room temperature. After washing again with 0.01 M PBS, the sections were placed in 1.5% DAB (3,3-diaminobenzidine) solution containing 0.02% H_2_O_2_ for color reaction. Finally, the sections were washed with 0.01 M PBS (pH 7.4) to stop the reaction. After the sections were dehydrated, permount coverslips were added.

### 2.8. Immunofluorescence Staining

Following three rinses in PBS, the sections were incubated in blocking medium containing 0.1% Triton X-100, 3% normal goat serum, and 2% bovine serum albumin for 1 h to block nonspecific binding. After blocking, the slides were incubated overnight in ubiquitin carboxyl terminal hydrolase 1 (UCH-L1) primary antibody (1:200, Santa Cruz, sc-271639). Alexa Fluor 488 goat anti-mouse IgG (1:200, Thermo Fisher, A-11029) secondary antibody was applied at room temperature for 60 min. After incubation in this secondary antibody, the slides were washed with PBS and mounted with 4′,6′-diamidino-2-phenylindole (DAPI) and Prolong Antifade Reagent.

### 2.9. Preparation of Protein (Protein Lysate) and Western Blot Analysis

All experimental tissues were collected in 0.01 M PBS, had protease inhibitors added (2 g/mL aprotinin, leupeptin, gasstatin A and 120 g/mL PMSF) and homogenized (Polytron RT MR3100). We collected the supernatant and added 3 µL standard and 150 µL protein analysis dye reagent (Bio-Rad, Hercules, CA, USA), diluted with distilled water (1:5), and measured the protein in 96-well plate (the absorbance at 595 nm on Bio-Tek Instruments). Then, an equal amount of protein was subjected to SDS gel electrophoresis and transferred to nitrocellulose (NC) paper (Trans-blot; BioRad). To explore the correlations of wound healing with oxidative stress and inflammation, we investigated the expressions of nuclear factor erythroid 2-related factor 2 (*NRF2*)-related antioxidant enzymes and inflammation-related proteins. Immuno-detection was applied with antibodies specific to NRF2 (1:1000, Affinity, AF0639), superoxide dismutase 1 (SOD1) (1:1000, Thermo, PA1–30195), glutathione peroxidase (GPx) (1:1000, Abcam, Ab22604), TGFβ-1 (1:500, Santa Cruz, sc-52893), MMP-2 (1:500, Santa Cruz, sc-13595), MMP-*9* (1:500, Santa Cruz, sc-13520), nuclear factor-κB (*NF*-*κB*, *p-65*) (1:500, Santa Cruz, sc-8008), UCH-L1 (1:500, Santa Cruz, sc-271639), and *VEGF* (1:500, Santa Cruz, sc-7269). Then, at room temperature with appropriate horseradish peroxidase (HRP), we conjugated the secondary antibody (1:5000, Sigma) to detect the immune signal. The following signals were observed by chemiluminescence (Renaissance kit; NEN, Boston, MA) and the signal intensity was quantified with Alphalmage 2000 (Alphalmage comp). β-actin (1:5000, Novus, NB600–501) was used to confirm the equal load of protein.

### 2.10. Statistical Analysis

SPSS 22.0 version statistical software (IBM Corporation, Armank, NY, USA) was used. One-way analysis of variance (ANOVA) and Student’s *t*-test were applied for determination of significance. Statistical significance was defined as *p* < 0.05.

## 3. Results

### 3.1. Rutin Improves Liver Function, Blood Lipid Profile, and Body Weight

**Figure 1:**
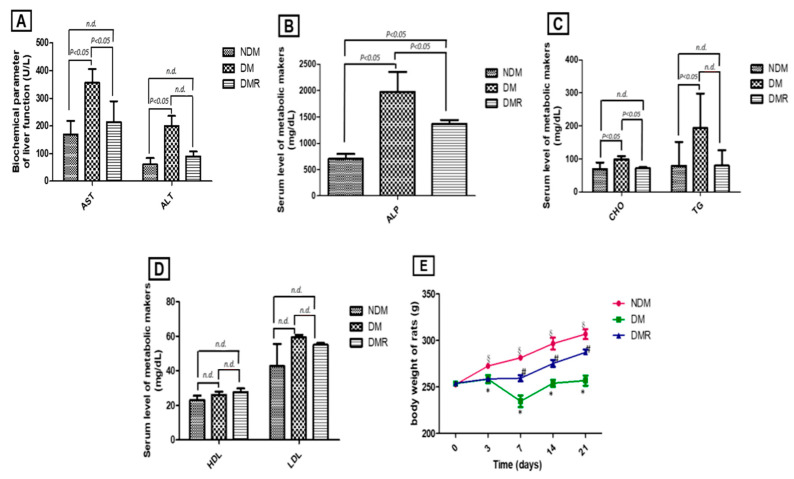
Histogram of serum biochemical markers and metabolic functions (**A**–**D**), including AST and ALT (**A**), ALP (**B**), CHO and TG (**C**), HDL and LDL (**D**). Line chart of 21 days of continuous observation of weight change (**E**). * *p* < 0.05 hyperglycemic (DM) compared with the normal (NDM) group, § *p* < 0.05 hyperglycemic with rutin (DMR) compared with the NDM group, # *p* < 0.05 DMR compared with the DM group, *n.d.* means no difference (*p* > 0.05).

ALP, AST, and ALT values were used to express liver function. In normal untreated rats, concentrations of AST, ALT (Figure 1A), ALP (Figure 1B), TG, and CHO (Figure 1C) were all within normal range. Following STZ treatment, impaired liver and metabolic functions were clearly demonstrated by enhanced AST, ALT, and ALP levels together with incidences of hypertriglyceridemia and hyperlipidemia. However, in the DMR group, both liver and metabolic functions gradually improved. There were no significant effects of STZ or rutin on HDL and LDL (Figure 1D). Body weight changes were observed for 21 consecutive days (Figure 1E). Rutin significantly ameliorated STZ-induced weight loss in hyperglycemic rats.

### 3.2. Rutin Improves Blood Sugar and Maintains Pancreatic Function in STZ-Induced Hyperglycemic Rats

**Figure 2:**
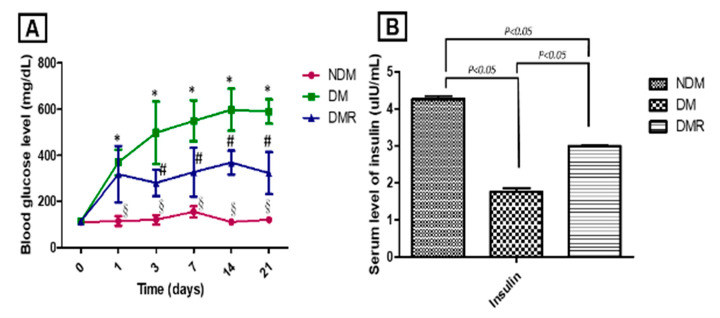
During the study period, blood glucose level (**A**) and serum insulin level (**B**) were monitored. * *p* < 0.05 DM compared with the NDM group, § *p* < 0.05 DMR compared with the NDM group, # *p* < 0.05 DMR compared with the DM group.

As shown in Figure 2A, STZ induces hyperglycemia (DM), while rutin effectively improves the hyperglycemia caused by STZ (DMR). The lowest serum insulin content was in the DM group (Figure 2B). Conversely, rutin maintained pancreatic function with higher serum insulin content in the DMR group than in the DM group.

### 3.3. Rutin Improves Wound Healing in STZ-Induced Hyperglycemic Rats

**Figure 3:**
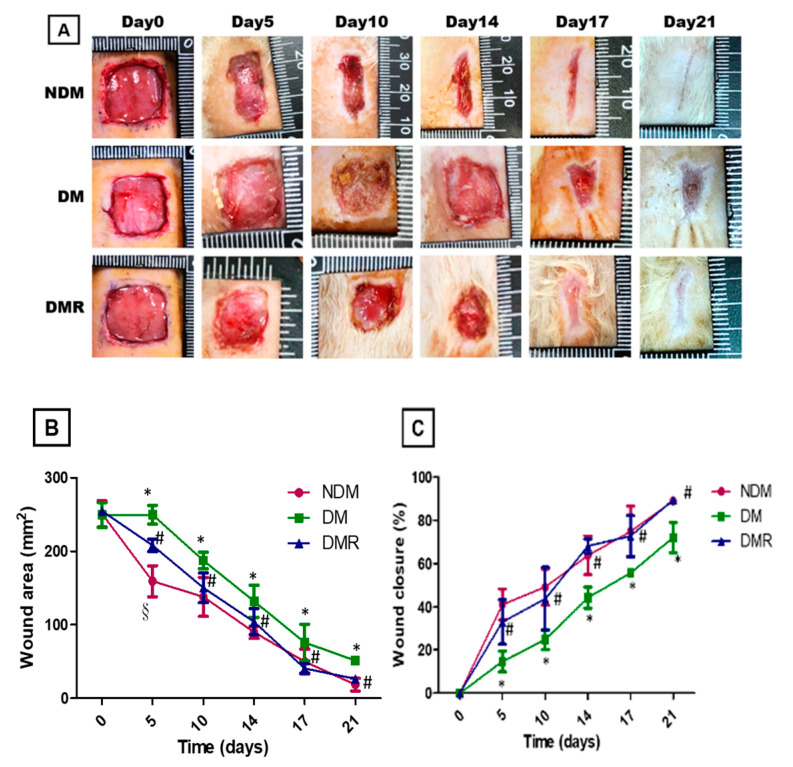
Continuous monitoring of 15 mm wound closure in each group over 21 days (**A**). The wound area (**B**) and wound closure (**C**) were calculated with NIH Image J analyzer by tracing the wound margin and calculating the pixel area. * *p* < 0.05 DM compared with the NDM group, § *p* < 0.05 DMR compared with the NDM group, # *p* < 0.05 DMR compared with the DM group.

Figure 3A shows the wound healing process in the NDM, DMR, and DM groups. Rats in the DM group showed impaired wound healing. Further, based on evaluation of wound edge and calculations of wound area (Figure 3B) and closure rate (Figure 3C), rutin improves wound healing in STZ-induced hyperglycemic rats.

### 3.4. Rutin Improves Proliferation of Collagen Fibers and Reduces Expression of Inflammatory Cells in the Wounds of STZ-Induced Hyperglycemic Rats

**Figure 4:**
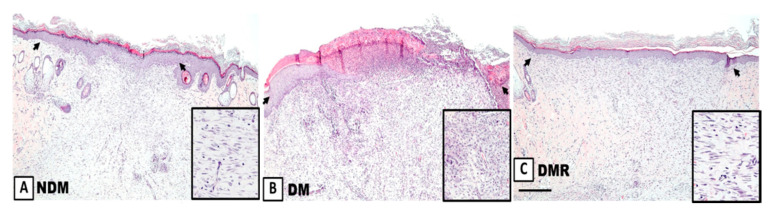
Representative images of wound tissue (**A**–**C**) on day 21 (H&E staining). The arrow indicates the width of re-epithelialization. The enlarged image shows the presence of inflammatory cells. Scale bar: 300 μm.

**Figure 5:**
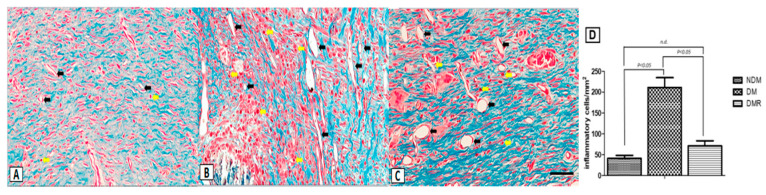
Masson’s trichrome staining of NDM (**A**), DM (**B**), and DMR (**C**) wound sections on day 21. There were significant strain-specific differences in collagen fibril deposition. The yellow arrow denotes inflammatory cells, while the black arrow denotes blood vessel. Quantitative analysis of inflammatory cells was performed using Image J analysis software (**D**). Scale bar: 50 μm.

Microscopic assessment with H&E staining showed that fibroblasts are the dominant cell type in the wound area on day 21. The enlarged image demonstrates that hyperglycemia promotes production of inflammatory cells, while rutin reduces the production of inflammatory cells (Figure 4). In terms of the effects of rutin on collagen deposition in wound sites, Masson’s trichrome staining showed increased collagen surrounding the fibroblasts in the granulation tissue on day 21 in all groups (Figure 5). Wounds in the DM group showed severe edema and disorganized pattern with heavy infiltration of inflammatory cells (Figure 5, yellow arrow). Wounds in the NDM and DMR groups demonstrated epidermal reorganization with complete restoration of normal wound microarchitecture. In addition, a large number of large-diameter blood vessels were observed in the DM group (Figure 5, black arrow). In contrast, the normal and rutin-treated groups demonstrated epidermal reorganization with complete restoration of normal wound microarchitecture without excessive increase in blood perfusion (Figure 4 and Figure 5).

### 3.5. Rutin Effectively Promotes NRF2, Targets Downstream Antioxidant Enzyme Activities, Suppresses Inflammation-Related Factors, and Facilitates Nerve Growth during Wound Healing

**Figure 6:**
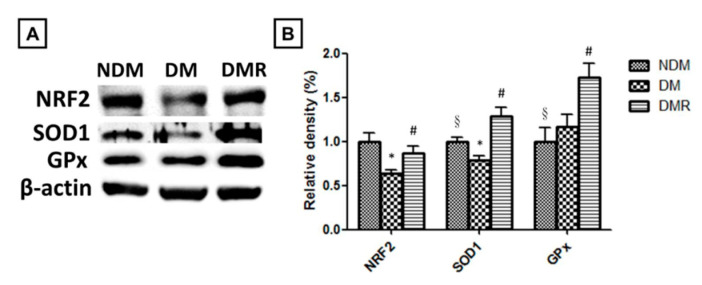
The expression of NRF2-related antioxidant enzymes was analyzed by Western blotting (**A**,**B**). * *p* < 0.05 DM compared with the NDM group, § *p* < 0.05 DMR compared with the NDM group, # *p* < 0.05 DMR compared with the DM group.

**Figure 7:**
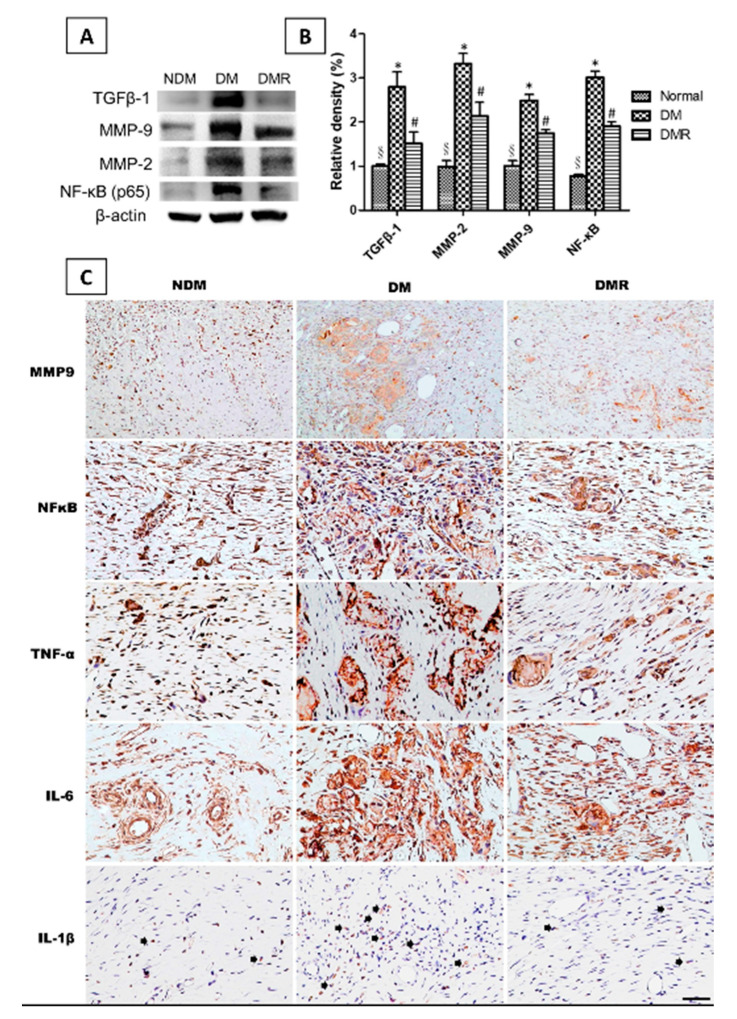
The expression of NF-κB, MMP-2, MMP-9 and TGFβ-1 was analyzed by Western blotting (**A**,**B**). Distributions of MMP-9, NF-κB, TNF-α, IL-6 and IL-1β (arrow) expressions based on immunohistochemistry results (**C**). Brown represents positive staining for MMP-9 in NDM, DM and DMR cytoplasms (magnification, ×100). * *p* < 0.05 DM compared with the NDM group, § *p* < 0.05 DMR compared with the NDM group, # *p* < 0.05 DMR compared with the DM group.

**Figure 8:**
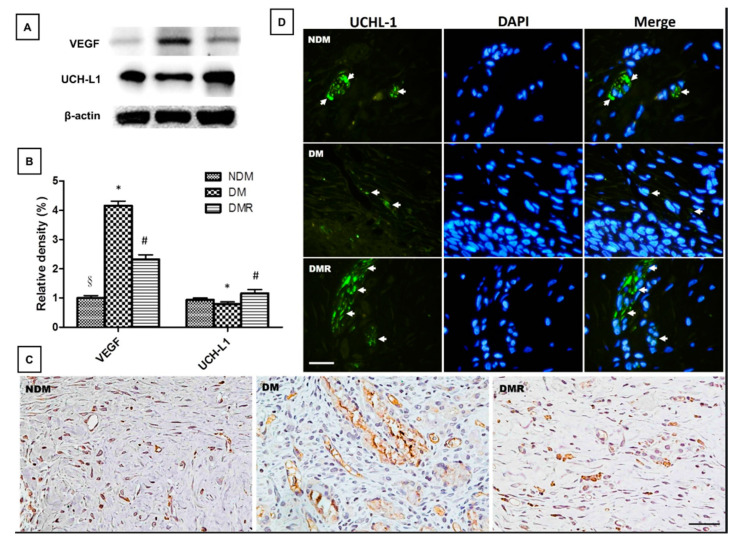
The expression of growth factors was analyzed by Western blotting (**A**,**B**). Distributions of VEGF (**C**) and UCH-L1 (**D**) expressions based on immunohistochemistry and immunofluorescence staining results. Brown represents positive staining for VEGF in NDM, DM and DMR cytoplasms (magnification, ×200). UCH-L1 immunofluorescence staining of wounds in NDM, DM, and DMR groups on day 21. Nerve fibers stained with UCH-L1 exhibited small single varicosities in DM group in the area of re-epithelialization. UCH-L1-immunoreactive nerve fibers in the NDM and DMR groups were expressed in cross and longitudinal sections of large nerve bundles. Arrows indicate UCHL-1 positive nerve fibers (green, UCH-L1-nerve fiber; blue, DAPI) (magnification, ×400). * *p* < 0.05 DM compared with the NDM group, § *p* < 0.05 DMR compared with the NDM group, # *p* < 0.05 DMR compared with the DM group.

Immunohistochemical and immunoblotting analyses were conducted to investigate whether treatment with rutin activates antioxidant enzymes and reduces inflammatory response of wounds in diabetic rats. The results of immunoblotting indicated that STZ reduces the production of antioxidant enzymes (i.e., SOD1 and GPx) related to NRF2. After rutin treatment, the activity of NRF2 and expressions of antioxidant enzymes in the wound significantly increased (Figure 6). Similar results were obtained for inflammation-related factors and growth factors. The immunohistochemistry and immunoblot data showed that STZ promotes the expressions of TGFβ-1, MMP-2, MMP-9, NF-κB, TNF-α, IL-1β, IL6, and VEGF and decreases the expressions of UCH-L1 (Figure 7 and Figure 8). After immunofluorescence staining, the UCHL1-labeled nerve fibers in the DM group were small and loose. Larger nerve bundles presenting with transverse and longitudinal sections were observed in the NDM and DMR groups (Figure 8D).

## 4. Discussion

Among the lower limb amputations performed in diabetic patients, more than 75% are due to DFU, which is currently the main cause of lower limb amputation without trauma [23]. However, the mortality rate 3 years after amputation is as high as 35% to 50% [24]. Poor glycemic control increases the risk of macrovascular disease, microvascular disease, neuropathy, and delayed wound healing. Similar results were obtained in this animal model study, with poor wound healing in STZ-induced hyperglycemic rats (Figure 3). Previous studies have shown that insulin produced by β cells is essential for maintaining glucose homeostasis and glucose is the most effective stimulator of insulin secretion [25]. The study demonstrated that flavonoids are potential substitutes for insulin secretagogues [25]. In addition, rutin has been shown to improve glucose homeostasis in streptozotocin-diabetic tissues by altering glycolysis and glycoisomerase [26]. Herein, we also confirmed that rutin treatment can reduce blood sugar in DMR rats (Figure 2). Insulin resistance caused by hyperglycemia promotes the pathogenesis of hyperlipidemia, but the underlying mechanism is still unclear [27]. Figure 1 shows that STZ induces AST, ALT, ALP, CHO and TG and that rutin has beneficial effects on AST, ALP, and CHO. There were no significant effects on HDL or LDL. A number of studies have shown contradictory relationships between glycemic control and HDL [28,29].

Cells and tissues in a hyperglycemic environment induce the generation of ROS, promote inflammatory response, and delay wound healing [30]. NRF2 prevents oxidative stress and regulates the production of related antioxidant enzymes, such as SOD1, GPx, heme oxygenase 1 (HO-1), and catalase [31,32]. The results of this study also confirmed that rutin increases the production of antioxidant enzymes SOD1 and GPx by upregulating the expression of NRF2 (Figure 6A). Recent studies have indicated that insufficient NRF2 leads to delayed wound healing and that proinflammatory cytokines are overexpressed in wounds [33]. Our results suggested a crucial role of NRF2 in promoting impaired healing process of diabetic wounds.

In addition to NRF2, NF-κB regulating antioxidant enzymes in DFU patients was also mentioned previously [30]. Both NRF2 and NF-κB transcriptionally mediated oxidative stress and triggered inflammation and affected one another [34]. The NF-κB family includes RelA (p65), RelB, c-rel, p50, and p52, with p50/p65 heterodimer being the most prominent [35]. Moreover, p65 has a negative effect on NRF2 through the expression of antioxidant response element (ARE)-related genes [34]. Figure 6B shows that rutin inhibits the expression of NF-κB (p65). Hyperglycemia can activate NF-κB and matrix-degrading enzyme MMPs [4], while activated NF-κB induces various inflammatory cytokines and MMPs [35,36]. It has been proposed that MMPs are essential to wound healing, with gelatinases (MMP-2 and MMP-9) involved in wound repair [37]. In this study, rutin reduced the inflammatory response via inhibition of the expressions of MMP-2 and MMP-9 (Figure 6B and Figure 7A). Furthermore, studies have suggested that TGFβ-1 stimulates the production of extracellular matrix molecules, and excessive TGFβ-1 and inflammatory cytokines may directly inhibit the expression of keratinocyte migration [38,39]. The results in Figure 7 also confirmed that rutin reduced TGFβ-1 and inflammatory cytokines (TNF-α, IL-6 and IL-1β).

A previous study has suggested that microvascular dysfunction and neuropathy are the main causes of wound healing difficulties in diabetic patients [40]. The process of wound healing is related to angiogenesis and neurogenesis and requires the participation of growth factors and proteins. In this study, staining of wound sections (Figure 4) showed that rutin promotes the regeneration of wound epithelium and that fibroblasts are regularly distributed. From the results of Masson’s trichrome staining (Figure 5), inflammatory cells are significantly reduced, and vascular structure is better in the wounds of DMR rats. Inflammatory cells produce cytokines and growth factors, which attract fibroblasts, promote cell migration and proliferation, and generate new blood vessels [41]. Angiogenesis refers to the restoration of blood flow to damaged tissues, providing oxygen and nutrients to repair cells [30]. VEGF is one of the most effective pro-angiogenic growth factors in the skin [30]. Rutin affects angiogenesis by reducing VEGF protein expression in the late stage of wound healing. Notably, cancer research has confirmed that MMP-9 triggers angiogenesis, especially VEGF [42]. Some drugs suppress VEGF by regulating the expression of MMP-9. The immunohistochemistry and immunoblot results of wounds demonstrated that rutin simultaneously decreases MMP-9 and VEGF (Figure 7 and Figure 8). This indicates that the anti-inflammatory process of rutin in wound healing is similar to that of cancer angiogenesis. In addition, the results of this study demonstrated that wounds are unable to heal normally if there is nerve supply failure [43]. UCH-L1 is a member of the protein ubiquitin hydrolase family, also known as protein gene product 9.5 (PGP 9.5) [44,45], and is strongly and persistently expressed in axons of peripheral neurons and cell bodies, making it an ideal neural marker to visualize the timing and extent of axonal projections in peripheral and visceral organs [46]. In this study, rutin induced expression of UCH-L1 protein in wounds of STZ-induced hyperglycemic rats (Figure 8). Experiments have indicated that rutin promotes wound neurogenesis, resulting in complete nerve innervation and more complete epithelial morphology.

## 5. Conclusions

From the results of this study, rutin significantly improved delayed wound healing in hyperglycemic rats. Rutin effectively up-regulates the expression of Nrf2 to increase the production of antioxidant enzymes and down-regulate the expression of NF-κB to reduce the production of MMPs, growth factors, and inflammatory cytokines in the wounds. The mechanistic representation of rutin for promoting wound healing may be derived from the regulation of blood sugar and excellent antioxidant and anti-inflammatory effects.

## Abbreviations

ALP, alkaline phosphatase; ALT, alanine aminotransferase; AST, aspartate aminotransferase; CHO, cholesterol; DFUs, diabetic foot ulcers; HDL, high-density lipoproteins; LDL, low-density lipoproteins; MMPs, matrix metalloproteinases; NF-κB, nuclear factor-κB; NRF2, nuclear factor erythroid 2-related factor 2; ROS, reactive oxygen species; TG, triglyceride; VEGF, vascular endothelial growth factor; UCH-L1, ubiquitin carboxyl terminal hydrolase 1.
